# The Laterality of Age-related Hearing Loss and Cognition

**DOI:** 10.1097/ONO.0000000000000008

**Published:** 2022-02-17

**Authors:** Alexander Chern, Alexandria L. Irace, Justin S. Golub

**Affiliations:** 1 Department of Otolaryngology—Head & Neck Surgery, NewYork-Presbyterian/Columbia University Irving Medical Center and Columbia University Vagelos College of Physicians and Surgeons, New York, New York; 2 Department of Otolaryngology—Head & Neck Surgery, NewYork-Presbyterian/Weill Cornell Medical Center, New York, New York.

**Keywords:** Age, related hearing loss, Cognition, Cognitive decline, Dementia, Hearing loss, Laterality, Presbycusis

## Abstract

**Objectives:**

To analyze the association between neurocognitive performance and age-related hearing loss in the right and left ear, individually.

**Design:**

Subjects included 5277 participants (≥50 years) from the general Hispanic population who underwent audiometric testing in a US multicentered epidemiologic study. Linear regression was performed to assess the cross-sectional association between cognitive performance (Digit Symbol Substitution Test [DSST], Word Frequency Test, Spanish-English Verbal Learning Test [SEVLT] 3 Trials, SEVLT Recall, and Six-Item Screener) and hearing in each ear (4-frequency pure-tone average), adjusting for age, sex, education, cardiovascular disease, and hearing aid use.

**Results:**

Mean age was 58.4 ± 6.2 years; 3254 (61.7%) were women. Mean pure-tone averages were 20.2 ± 11.7 dB (right ear) and 20.2 ± 12.3 dB (left ear). Multivariable regression demonstrated significant associations between all cognitive tests and hearing loss in both ears.

**Conclusions:**

Worsening hearing loss in the right and left ear was associated with decreased performance across all tests. No laterality in the association was demonstrated.

Age-related hearing loss (ARHL) is a common and undertreated condition of older life. Recent studies have demonstrated an independent association between hearing loss (HL) and cognitive decline.(1,2) A recent summary report found HL to be the largest modifiable risk factor for dementia.(3)

One hypothesized mechanism explaining this association is that HL causes functional (4) and structural (5) brain changes. These changes may have downstream effects on cognitive processes, predisposing individuals to risks of cognitive decline and dementia.

Herein, we explore whether auditory laterality (left-right ear differences) contributes to the effect of ARHL on the brain. ARHL usually presents as bilateral, high-frequency, progressive sensorineural HL associated with aging. However, ARHL is not always perfectly symmetric and can present with modest asymmetries (particularly in the older old population).(6) Other studies have shown that HL is associated with lateralized structural changes in right and left hemispheres of the brain (7,8) while others suggest the left and right ear provide asymmetric input along ascending auditory pathways.(9,10) Indeed, each ear has stronger neural connections to the contralateral hemisphere (11) suggesting that HL in each ear may be associated with varying central findings (ie, on neuroimaging) and clinical manifestations (ie, domain-specific cognitive performance). Dichotic listening tests often show a right-ear advantage given stronger connections between the right ear and the typically dominant left-sided auditory cortices.(12,13)

To our knowledge, previous studies have not investigated the association between cognition and laterality of HL. Our objective was to perform cross-sectional analyses examining neurocognitive performance and ARHL in the right and left ear.

## MATERIALS AND METHODS

### Study Cohort

The Hispanic Community Health Study/Study of Latinos is a multicentered, prospective epidemiologic cohort study.(14) Subjects with pure-tone audiometry and neurocognitive testing (from the one available 2008–2011 wave) were included; assessments were performed in English or Spanish per subject preference. Subjects with missing records, audiometric data, covariate data, or cognitive testing were excluded. Subjects <50 years or with early-onset HL were also excluded to limit subjects to those at risk for ARHL.

### Exposure Variables

Hearing was assessed with pure-tone audiometry; hearing thresholds (dB, decibel hearing level) were measured from low (500 Hz) to high (8000 Hz) frequency. Worse hearing was indicated with higher hearing thresholds, while better hearing was indicated with lower hearing thresholds. Right ear hearing and left ear hearing were the primary exposure variables, defined as the four-frequency pure-tone average (PTA) of the right and left ear, respectively. The PTA was defined as the mean hearing threshold (in dB) at 500, 1000, 2000, and 4000 Hz. HL severity was categorized as follows: normal hearing (0–25 dB), mild HL (26–40 dB), moderate HL (41–55 dB), moderately-severe HL (56–70 dB), severe HL (71–90 dB), and profound HL (≥91 dB). Because few participants had severe or profound HL, these categories were combined with the moderately-severe HL group (PTA ≥56 dB category of moderately-severe or worse hearing).

### Outcome Variables

Main outcome variables were behavioral tests designed to assess a wide range of neurocognitive function. These cognitive tests (and cognitive domains they assess) included Digit Symbol Substitution Test (DSST; speed and attention), Word Frequency Test (verbal fluency), Spanish-English Verbal Learning Test (SEVLT [3 Trials and Recall]; verbal memory and learning), and Six-Item Screener (global cognitive function). Higher scores indicated better cognitive performance. Justification for employing specific assessments are found in previous study by our group.(2)

### Covariates

Variables that may confound the association between HL and cognitive performance were included in our multivariate analysis as covariates. These covariates included age (years), sex (male/female), education (total years of schooling), hearing aid use (yes/no), and cardiovascular disease (composite score from 0 to 5; higher score indicates worse disease). A composite cardiovascular disease score was created from several risk factors to avoid multicollinearity in our models.

### Statistical Analysis

Continuous variables were described with means and standard deviations; categorical variables were described with frequencies and proportions. Multivariable linear regression models were employed to examine the association between each assessment of neurocognitive performance and hearing in the right and left ear, adjusting for covariates. Results were reported in the form of 10-dB change in PTA (equivalent to a half-category change in hearing level). Statistical analysis was performed using R 3.6.3 (R Foundation for Statistical Computing) with RStudio 1.2.5033 (RStudio, Inc, Boston, MA). *P* values were considered statistically significant at *P* < 0.05 (2-tailed); estimates were described using 95% confidence intervals (CI).

The study was designated not human subjects research under 45 CFR 46 (AAAQ9546(M00Y01) by the Columbia University Institutional Review Board.

## RESULTS

### Demographics

A total of 16,415 subjects were included in the original study cohort. Subjects with missing records, audiometric data, covariate data, or cognitive testing were excluded. Subjects <50 years or with early-onset HL were also excluded to limit subjects to those at risk for ARHL. Among 5277 individuals who remained for analysis after applying exclusion criteria, mean (standard deviation) age was 58.4 (6.2) years, 3254 (61.7%) were women, 46 (0.9%) used hearing aids, mean education level was 10.5 years, mean composite cardiovascular score was 1.7. Mean scores of cognitive tests are listed in Table 1.

**TABLE 1.** Baseline characteristics of subjects

Baseline characteristic	Total cohort (n = 5277)		
Age, y, mean ± SD	58.4 ± 6.2		
Women, no., %	3254 (61.7)		
Hearing aid use, no., %	46 (0.9)		
Education, y, mean ± SD	10.5 ± 4.6		
Composite cardiovascular disease score,^*a*^ mean ± SD	1.7 ± 1.1		
DSST score, mean ± SD	32.1 ± 13.0		
Word Frequency Test score, mean ± SD	18.1 ± 7.2		
Six-Item Screener score, mean ± SD	5.3 ± 0.9		
SEVLT 3 Trial score, mean ± SD	22.4 ± 5.5		
SEVLT Recall score, mean ± SD	8.1 ± 2.9		
	Better hearing ear (n = 5277)^*b*^	Right ear (n = 5190)	Left ear (n = 5193)
Normal hearing, no., %^*c*^	4347 (82.4)	3997 (77.0)	3953 (76.1)
Mild HL, no., %	746 (14.1)	918 (17.7)	954 (18.4)
Moderate HL, no., %	136 (2.6)	191 (3.7)	198 (3.8)
Moderately-severe or worse HL, no., %	48 (0.9)	84 (1.6)	88 (1.7)

^*a*^Cardiovascular disease score ranges from 0 (lowest) to 5 (highest). Points were assigned for each component (1 for coronary artery disease, 1 for hypertension, 1 for stroke; 1 for impaired glucose tolerance, 2 for diabetes).

^*b*^The sample size for right ear and left ear is slightly largely than the total cohort because there were 87 participants that were missing right ear pure-tone audiometry, as well as a separate 84 participants that were missing left ear pure-tone audiometry.

^*c*^Hearing loss was defined as pure-tone average >25 dB.

DSST indicates Digit Symbol Substitution Test; HL, hearing loss; SD, standard deviation; SEVLT, Spanish-English Verbal Learning Test.

Mean PTA was 20.2 (11.7) dB in the right ear and 20.2 (12.3) dB in the left ear. The breakdown of subjects in each HL category is listed in Table 1. There was no significant difference between PTA in the right ear versus left ear (*P* = 0.84).

### Multivariable Linear Regression Models

Multivariable regression demonstrated significant associations between all cognitive tests and age-related HL in both the left and right ear (Table 2). For every 10-dB worsening in right ear PTA, there was a significant decrease in DSST (1.22-point decrease, 95% CI, 0.7-1.74), Word Frequency Test (0.62, 0.28-0.95), SEVLT 3 Trials (0.64, 0.39-0.9), SEVLT Recall (0.41, 0.27-0.55), and Six-Item Screener scores (0.05, 0.01-0.1). For every 10-dB worsening in left ear PTA, there was a significant decrease in DSST (1.12, 0.61-1.62), Word Frequency Test (0.76, 0.44-1.08), SEVLT 3 Trials (0.67, 0.43-9.92), SEVLT Recall (0.40, 0.26-0.53), and Six-Item Screener scores (0.06, 0.02-0.1).

**TABLE 2.** Multivariable regression models for cognitive performance and laterality of hearing loss

Neurocognitive assessment	Multivariable linear regression^*a*^ (right ear PTA)^*b*^		Multivariable linear regression^*a*^ (left ear PTA)^*b*^	
	Score decrease per 10 dB worsening in hearing (95% CI)	*P*	Score decrease per 10 dB worsening in hearing (95% CI)	*P*
DSST	1.22 (0.7-1.74)	<0.001^*c*^	1.12 (0.61-1.62)	<0.001^*c*^
Word Frequency Test	0.62 (0.28-0.95)	<0.001^*c*^	0.76 (0.44-1.08)	<0.001^*c*^
SEVLT 3 Trials	0.64 (0.39-0.9)	<0.001^*c*^	0.67 (0.43-9.92)	<0.001^*c*^
SEVLT Recall	0.41 (0.27-0.55)	<0.001^*c*^	0.40 (0.26-0.53)	<0.001^*c*^
Six-Item Screener	0.05 (0.01-0.1)	0.011^*c*^	0.06 (0.02-0.10)	0.003^*c*^

^*a*^Multivariable models were conducted adjusting for covariates, including age, gender, education, hearing aid use, and cardiovascular disease.

^*b*^Hearing was defined by the PTA.

^*c*^Significant, *P* < 0.05.

CI indicates confidence interval; DSST, Digit Symbol Substitution Test; PTA, pure-tone average; SEVLT, Spanish-English Verbal Learning Test.

Across all cognitive tests, 95% CIs highly overlapped between left ear PTA-cognition associations and right ear PTA-cognition associations. Thus, there was no observed evidence of a difference between laterality of hearing and cognitive performance.

## DISCUSSION

A growing body of evidence has demonstrated an independent association between HL and cognitive performance. Previous studies have examined the laterality of HL and its associated volumetric brain changes.(7) As such, the objective of our study was to investigate the role of auditory laterality in the relationship between HL and neurocognitive performance. To our knowledge, no current literature has explored this topic. Our results show that worse hearing in both the right and left ear were both and similarly associated with decreased cognitive performance measured by all neurocognitive tests.

Neuroimaging studies have demonstrated associations between HL with both structural and functional brain changes.(15) These changes may increase risk of cognitive decline and dementia. One recent study found that poorer right-sided, but not left-sided, hearing in midlife was related to a faster decline in volumes of both temporal lobes.(7) Furthermore, studies in dichotic listening tests have shown a right ear advantage.(12,13) Right-sided hearing has been associated with improved verbal stimuli (12) and could be more strongly related to language and memory. This may be explained by stronger central connections between the right ear and the typically dominant left temporal lobe language centers. Given previously observed associations between ARHL and cognition, this may suggest that those who have slightly worse right-sided ARHL may be at a relatively increased risk for worse cognition. Alternatively, left ear dysfunction may be more impactful in downstream effects on cognition; one study found that left-ear dichotic listening scores were directly proportional to global brain connectivity in subjects with Alzheimer’s disease.(13) As suggested by our findings, it is also plausible that relative contributions by the right and left ear to the association between ARHL and cognitive performance are negligible.

Our study had limitations. Our study focused on an understudied (Hispanic/Latino) population, limiting its generalizability. However, most prior research has neglected minoritized populations such as Hispanics. Auditory laterality may contribute to asymmetric brain changes in regions responsible for various cognitive domains; however, changes may not be detectable using our cognitive tests. Laterality of the dominant auditory cortex was not taken into account. Employing neuroimaging or domain-specific neurocognitive tests may help localize brain effects of side-specific differences in HL. Future studies should investigate if widely asymmetric/unilateral HL and other measures of HL (speech recognition, speech-in-noise) are more strongly associated with cognitive decline and dementia. These investigations may help elucidate mechanistic understanding of laterality contributions to cognitive decline and dementia. Although any relationship between ear laterality and cognition would not be fully explained by pure-tone audiometry alone, our study presents a compelling initial exploration into this given the well-established association between HL (measured by pure tone audiometry) and cognition.(15)

## CONCLUSION

Worsening HL in both right and left ears was associated with decreased performance on several neurocognitive assessments, adjusting for confounders. No auditory laterality contributing to the association between ARHL and decreased neurocognitive performance was found. Our findings will help inform understanding of the laterality of auditory processing.

## FUNDING SOURCES

None declared.

## CONFLICT OF INTEREST

Justin S. Golub received travel expenses for industry-sponsored meetings (Cochlear, Advanced Bionics, Oticon Medical), consulting fees or honoraria (Oticon Medical, Auditory Insight, Optinose, Abbott, Decibel Therapeutics, Alcon), department received unrestricted educational grants (Storz, Stryker, Acclarent, 3NT, Decibel Therapeutics).

## DATA AVAILABILITY

The data that support the findings of this study are openly available by request from the Hispanic Community Health Study/Study of Latinos at https://sites.cscc.unc.edu/hchs/.
